# Restricted and repetitive behaviors and their developmental and demographic correlates in 4–8-year-old children: A transdiagnostic approach

**DOI:** 10.3389/fnbeh.2023.1085404

**Published:** 2023-03-01

**Authors:** Jennifer Keating, Stephanie Van Goozen, Mirko Uljarevic, Dale Hay, Susan R. Leekam

**Affiliations:** ^1^Cardiff University Centre for Human Developmental Science, School of Psychology, Cardiff University, Cardiff, United Kingdom; ^2^Department of Psychiatry and Behavioral Sciences, Stanford University, Stanford, CA, United States

**Keywords:** restricted and repetitive behaviors (RRB), repetitive sensory and motor behaviors, insistence on sameness, transdiagnostic, anxiety, developmental correlates, demographic correlates

## Abstract

**Background:** Restricted and repetitive behaviors (RRBs) are a broad class of behaviors characterized by frequent action repetition and intense preference for sameness. Research has predominantly focused on RRBs in diagnosed clinical groups, particularly in autism spectrum disorder and genetic disorders. Using a transdiagnostic approach, the current study examined RRBs in a diverse sample of children in relation to developmental and demographic correlates (age, language, non-verbal ability, child anxiety, sex, and socioeconomic status). Separate analyses examined two RRB subtypes; repetitive sensory and motor behaviors (RSMB) and insistence on sameness (IS).

**Method:** Children (*N* = 260, age 4–8 years, 174 male, 86 female) in mainstream schools identified by teachers as having behavioral, emotional, and/or cognitive difficulties, were assessed using the Repetitive Behavior Questionnaire-2 (RBQ-2), the British Picture Vocabulary Scale (BPVS), Lucid Ability Scale, the Welsh Index of Multiple Deprivation (WIMD) and the Screen for Child Anxiety Related Emotional Disorders (SCARED). Recruitment excluded diagnosed clinical conditions. The Strengths and Difficulties Questionnaire (SDQ) was used to assess children’s difficulties.

**Results:** RRB scores were of high frequency and the scores for the IS were higher than for RSMB. The severity of anxiety symptoms and male sex were significantly associated with both RRB subtypes, and younger age and SES scores were associated with IS. Elevated RRB total and subtype scores were significantly related to SDQ scores for emotion, conduct, hyperactivity, and peer-relations.

**Discussion:** The study provides the first evidence of RRBs in a diverse sample of young children with emerging difficulties in behavior, cognition, and/or emotion. The results contribute to proposals about psychological development in RRB and indicate that RRBs are best represented on a continuum of severity found across children in the early school years. The results support previous findings of a relation between RRB and anxiety reported in clinical samples and importantly, they indicate that it is time to move beyond the study of categorically defined groups and consider correlates of RRBs that include broad indices of mental health and well-being.

## Introduction

Restricted and repetitive behaviors (RRB) form a broad class of behaviors that are characterized by frequent action repetition and an intense preference for sameness. Restrictedness is apparent in the narrowness of focus, inflexibility in interests, and activities and insistence that aspects of the environment stay the same. Repetition is manifested in rhythmic motor stereotypies, repetitive speech, routines, and rituals (Leekam et al., 2011). Factor analysis studies that include both clinical and normative samples have frequently summarized RRBs into two subtypes: repetitive motor behaviors (RMB) which often includes a sensory element (RSMB) and insistence on sameness (IS; Bishop et al., 2013; Evans et al., 2017). Some studies, for example, those using The Repetitive Behavior Scale-Revised (RBR-R), have found more than two subtypes (e.g., Lam and Aman, 2007; Kästel et al., 2021). However a recent meta-analysis of all RRB factor analytic studies until 2022 (Uljarević et al., in press), reported that the two RMB and IS factors were the most consistent factors that emerged across all studies using any of nine different dedicated RRB measures. Furthermore, relevant to the current study, RMB and IS were two key factors that consistently emerged across different time points during early normative development (Uljarević et al., 2017; Sifre et al., 2021). The current study, therefore, focused on these two factors to investigate developmental and demographic correlates of RRB in young children.

Although RRBs are found in the general population, research has predominantly focused on exploring and characterizing the presentation and correlates of the most high-frequency RRBs found in diagnosed clinical groups, particularly autism spectrum disorder (ASD; Uljarević et al., in press). While this focus gives informative insights into ASD, it gives less insight into the broader nature of RRB itself, because all individuals with an ASD diagnosis have a particular pattern of RRBs, including type or form, quantity, and high-intensity and their RRBs always co-occur with social communication difficulties. This means that the full variation of RRBs and the independence of these behaviors from other symptoms is difficult to clarify. Likewise, while RRBs are also found in a range of other genetic and clinical conditions; Prader-Willi syndrome, Williams syndrome, Fragile X syndrome, Angelman’s syndrome, Cri de Chat syndrome, Down syndrome, Lowe syndrome, Smith-Magenis, PTEN mutations, 22q11.2 deletion syndrome, obsessive compulsive disorder (OCD), Tic disorders, eating disorders, psychotic disorders, Attention deficit hyperactivity disorder (ADHD), RRBs often form defining diagnostic symptoms of these conditions or there is often a high incidence of overlapping diagnosis with ASD. To better understand the broader presentation of RRBs across populations and their relation to other factors, different samples of children in non-clinical populations need to be studied irrespective of their inclusion due to RRB diagnostic criteria.

Taking a transdiagnostic approach (Cuthbert, 2014; Astle et al., 2022), the goal of the current study was to set aside sample selection by diagnostic category and describe RRBs in a diverse non-clinical sample of 4–8-year-old children. Study inclusion was based on “functional” recruitment (see Astle et al., 2022), in this case, the functional need for assessment due to behavioral, cognitive, or emotional difficulties that had been identified at school. For the current study, none of the children had a clinical diagnosis at the time of referral, avoiding the circularity of selecting children with specific types of elevated RRBs as part of their diagnosis. In this respect, the study differs from a group-based design comparing those with a diagnosis to those without. Although the children may be at risk for a range of different psychological problems and although some children in time might come to gain a diagnostic label, their inclusion in the study is not defined by the presence of RRB symptomatology. Likewise, the study also differs from previous studies of typical development which include a “neurotypical” or “no diagnosis” group. The children in the current sample have not been screened as being free of a diagnostic condition; nor are they equivalent to the children included in community sample research designs in that they are likely to have a heightened risk for psychological problems of some kind.

The first aim was to describe the variation of RRBs in this sample against the background of known levels of RRB reported in the literature. We used the results from a community sample (Uljarević et al., 2017) as a comparative benchmark as this sample included the same age group used in the current study. The second aim was to explore the contribution to RRBs made by a range of developmental and demographic variables, each of which have been previously identified as correlates of RRBs in either clinical samples, particularly ASD groups, or in community samples. The developmental variables included age, language, non-verbal ability, and child anxiety and the demographic variables included sex and socioeconomic status. While each variable has previously been tested in previous studies, few, if any studies have systematically assessed all these variables together (see Uljarević et al., in press, for review).

Understanding RRBs in the context of children’s development may help to clarify the contribution of several factors to RRBs. According to developmental psychological accounts (Thelen, 1981; Evans and Gray, 2000; Leekam et al., 2011), RRBs are universal in infancy. Early motor and sensory RRBs provide an adaptive function for neural maturation and for motor, cognitive and emotional domains of development but this changes as goal-directed self-regulation increases; thus, high levels of RRBs gradually reduce as behaviors come under greater voluntary control (Thelen, 1979, 1981). It has been proposed that excessive levels of RRBs maintained later in childhood may represent developmentally immature responses maintained within the behavioral repertoire at an age when they are no longer developmentally adaptive (Evans et al., 1999; Leekam et al., 2011). For example, in the motor and cognitive domain, it is proposed that high levels of RRB early in development facilitate neuromuscular skills, release motor tension, and regulate arousal, but become less intense in frequency with the onset of cognition and language, due to increasing cognitive and verbal regulation and alternative self-guided action selection (Thelen, 1981). In the emotion domain, it is proposed that high levels of routines and “just right” behaviors are recruited as means to ward off common fears and anxiety that specifically develop in the early years (Evans et al., 1999; Uljarević and Evans, 2017), but become less intense as cognitive and emotion-regulation strategies develop with age. Therefore, psychological development involving regulatory mechanisms within motor, cognitive-linguistic, and emotional domains, may be related to immature levels of repetitive behavior.

Evidence in support of a developmental account of RRBs has been found in studies of both typical development and studies of children with an ASD diagnosis. First with respect to the domains of cognition and language, results from community samples show that RRBs reduce as children become more cognitively and linguistically skilled with age. For example, in a longitudinal study, Larkin et al. (2017) found a decline in the frequency of the RSMB of RRB. This decline was specifically associated with improvements in language and cognitive ability in young children from two to five years. Harrop et al. (2014) and Ray-Subramanian and Weismer (2012) also reported that sensory and motor repetitive behaviors were negatively correlated with typically developing 2- and 3-year-old language and cognitive skills. Studies of groups with ASD-and genetic conditions are difficult to compare directly with community sample results as they tend to include wide age ranges and lower IQ levels, but these studies show broadly similar trends. For example, non-verbal IQ was negatively associated with both RSMB and IS aspects of RRBs in a large sample of autistic children aged 15 months to 11 years (Bishop et al., 2006). Non-verbal IQ was also negatively associated with the restricted interests subscale of the RBS-R in a sample of boys aged 6–10 years with Fragile X syndrome (Oakes et al., 2016). Also, in a longitudinal study of autistic children from ages 6–11 years, difficulty with routine change was associated with both age and non-verbal IQ, such that for children with lower non-verbal IQ, these difficulties became more prevalent over time, while children with higher initial non-verbal IQ remained relatively stable over time (Courchesne et al., 2021). Therefore, in the current sample, we might expect to see associations with age, verbal and/or non-verbal skills, such that lower age, verbal, or non-verbal skills will be associated with higher incidences of RRBs.

Second, with respect to the domain of emotion development, research findings show that high levels of fear and anxiety are associated with high levels of RRBs. Studies of typical development have reported a significant relation between routines, rituals, or compulsions and high levels of anxiety or worry in 7–16-year-olds (Laing et al., 2009) and between sensory RRBs and childhood fears in children with a mean age of 4-years (Uljarević and Evans, 2017). Anxiety, however, is an extensively documented associate of RRBs in the ASD literature (see Sellick et al., 2021 for review) with research suggesting that repetitive behavior severity is an early indicator of risk for elevated anxiety symptoms in autism spectrum disorder (Baribeau et al., 2020). Anxiety-RRB associations have also been reported in other diagnosed groups including Down syndrome (Uljarević and Evans, 2017), Fragile X syndrome (Lozano et al., 2022), 22q11.2 (Uljarević et al., 2019), and in individuals with *PTEN* mutations independent of ASD (Uljarević et al., 2022), although were not found in individuals with Williams syndrome (WS) even though the WS sample had elevated anxiety (Rodgers et al., 2012a).

It has also been proposed that the relation between RRB may be specific to one subtype of RRB; that of routines and intense preference for sameness (IS) rather than to the RSMB subtype (Baribeau et al., 2020). However, there is insufficient research evidence to confirm this. While it is true that an IS-anxiety association has been extensively reported (e.g., Gotham et al., 2013; Baribeau et al., 2020), to our knowledge only two studies have tested its selective nature by including not only IS items in the study but also RSMB items and testing for a difference (Rodgers et al., 2012b; Lidstone et al., 2014). Therefore, in the current study, we predict an association between RRB and anxiety, given previous evidence, but no specific predictions are made that the RRB-anxiety association will be selective to IS. High levels of anxiety and emotion have already been reported in the current sample when different research questions have been investigated (Adegboye et al., 2021, 2022; Howe-Davies et al., 2022). However, no previous studies have explored the relation between anxiety and repetitive behavior in a sample of this kind.

In addition to developmental variables, including cognition, language, and emotion, several demographic variables have also been linked to RRBs in children although the evidence is mixed. With respect to SES there is some evidence in community population samples of RRB associations with SES (Leekam et al., 2007; Larkin et al., 2017), although there are few replications with SES as an included variable. In terms of the effects of sex, a recent review of the ASD literature reported 11 studies that examined the relation between sex and RRB subscales and concluded that most studies did not find a significant association. In the few studies in which sex effects are found, however, it is males that have higher levels of RRB (Uljarević et al., in press). Therefore, no specific predictions are made for SES or sex.

Finally, given that children were recruited to this sample due to emotional, behavioral, and cognitive difficulties at school, the study offered an opportunity to explore the relation between restricted and repetitive behavioral responses and other aspects of general mental health in addition to anxiety. While several studies focusing exclusively on ASD-diagnosed children have found a relation between elevated RRBs and hyperactivity (Gabriels et al., 2005; Tsai et al., 2020), very few previous studies to date have studied RRBs and children’s behavioral and emotional difficulties in samples without a neurodevelopmental diagnosis. One previous study (Ghanizadeh and Moeini, 2011) to our knowledge has examined this relation in a typical preschool community sample, using the Strengths and Difficulties Questionnaire (SDQ), a questionnaire widely used to screen for child mental health (Goodman et al., 2000; Goodman, 2001). This study found moderate correlations between RRB subscales and the SDQ subscales of emotion, conduct, hyperactivity, and peer relations. Emotion and hyperactivity subscales had the strongest correlations. The current study aimed to replicate this investigation for the first time in a sample selected with behavioral, emotional, and/or cognitive difficulties. The SDQ profiles of this sample have already been reported in several studies (Adegboye et al., 2021, 2022) and are characterized by high scores on all subscales. If RRB scores are also associated with heightened scores on subscales of the SDQ, this opens new interpretations for understanding the broader presentation of RRBs, especially in light of the possible adaptive or maladaptive functions proposed by developmental accounts of RRB.

In summary, the aim of this study was to use a transdiagnostic approach to describe the pattern of RRBs and their correlates in a diverse sample of children at risk for a range of different psychological problems but whose inclusion in the study is not defined by the presence of RRB symptomatology. Given the developmental theory of RRBs and supporting research evidence from previous clinical and non-clinical studies, we predicted that RRBs would be associated with developmental skills in the domains of cognition, language, and emotion. Emotion was specifically assessed through a child anxiety measure but the current study additionally explored the association between RRB and other broader indicators of mental health and well-being.

## Materials and methods

### Participants

The sample comprised 260 4–8-year-olds referred to Cardiff University’s Neurodevelopment Assessment Unit (NDAU^1^). There were 103 children aged 4–5 years-old and 157 children aged 6–8 years of which 11 were aged 8. None of the children had a clinical diagnosis of neurodevelopmental and/or learning disorders at the time of testing. Children (aged 4–8 years) were referred for NDAU assessment by teachers and Special Educational Needs Coordinators (SENCOs) as having emotional, cognitive, and/or behavioral difficulties in the classroom. All referrals were made by local mainstream schools in the area. The NDAU assessment unit is not a clinical unit. It provides detailed assessments of the child across different psychological domains—cognition, language, emotion, etc. in line with the approach of the Research Domains Criteria (RDoC) framework (Cuthbert, 2014), it is not concerned with conventional clinical diagnoses (e.g., DSM-5) Instead, its goal is to understand the patterns of psychological functioning and behavior shown by children for the purpose of informing research and helping the school to understand each child’s profile. While the processes investigated may have relevance to different diagnostic categories, the remit of the unit is not to diagnose or to establish whether children eventually receive a clinical neurodevelopmental diagnosis. Demographic details of the sample are shown in Table 1. One-hundred and seventy-four of the children were male and 86 were female. 43.4% of the sample had low SES as indicated by being within the two highest quintiles of the Welsh Index of Multiple Deprivation (WIMD; Welsh Government, 2019).

**Table 1:** Characteristics of sample, age, sex, SES, verbal, and non-verbal ability and anxiety.

	Mean (SD), range, or percentage	Range
Age		
Months, mean (SD), range	75.19	49–100
Percent aged 4–5 years	39.6%	-
Percent aged 6–8 years	60.4%	-
Sex		
Percent male,	66.9	-
Percent female	33.1	-
SES		
WIMD Rank, mean (SD), range	905.77 (574.53)	12–1902
WIMD Quintiles, mean (SD), range	2.88 (1.46)	1–5
Percent in the two most deprived quintile categories	43.4%	-
Verbal ability		
BPVS Standard	94.11 (12.05)	63–128
Percent below-average scores	20.9%	-
Lucid Verbal Reasoning	104.66 (14.92)	62–190
Percent below-average scores	6.4%	-
Non-verbal ability		
Lucid Non-Verbal Reasoning	94.04 (17.15)	60–150
Percent below scores	29.4%	-
Anxiety score		
SCARED, mean (SD), range	19.92 (14.84)	0–70
Percent score of ≥ 25.	30.1	-

*Note: SES, social-economic status; WIMD, Welsh Index of Multiple Deprivation 2019 data. Available online at: https://statswales.gov.wales/Catalogue/Community-Safety-and-Social-Inclusion/Welsh-Index-of-Multiple-Deprivation; BPVS, British Picture Vocabulary Scale*.

### Procedure

Data collection took place between September 2017 and September 2021. Consecutive referrals are reported. However, RRB data were not available for 38 children due to a data entry error, and testing at NDAU was paused from March-September 2020 (due to Covid-19 lockdown). At the visit to NDAU, each child was given a battery of task-based assessments and their parents/guardians completed questionnaire measures. The research procedures were approved by Cardiff University’s Ethics Committee (EC.16.10.11.4592GR). Parents/guardians gave written informed consent on behalf of the child and the child gave their assent.

### Measures

#### The repetitive behavior questionnaire-2

The RBQ-2 (Leekam et al., 2007) was selected instead of other established measures (e.g., Bodfish et al., 2000; Le Couteur et al., 2003) because of its suitability for a diverse child sample. The RBQ-2 was originally developed and tested in a normative longitudinal sample of children aged 15 months to 6 years (Leekam et al., 2007; Larkin et al., 2017) and later published for individuals diagnosed with ASD (Lidstone et al., 2014). The questionnaire is completed by a parent/guardian. It consists of 20 items, scored 1, 2, or 3 (never/ rarely, mild/occasional, or marked/notable). Items include motor behaviors (e.g., rocking, repetitive hand/finger movements), sensory behaviors (e.g., special interest in the feel of surfaces), restricted interests (e.g., playing the same music, game, or video), and routines (e.g., insisting that aspects of daily routine must remain the same). Parents are asked to rate behaviors shown in the previous month. Higher scores represent an increased level of, and/or impact of the RRB. The RBQ-2 has a stable two-factor structure assessed using items 1–19. These are: (1) repetitive sensory and motor behavior (RSMB); and (2) routines-rituals-restricted interests. The routines-rituals-restricted-interests subscale is often referred to inclusively as “insistence on sameness” (IS). The two subscales have excellent internal consistency in samples of very young (Leekam et al., 2007), and older (Uljarević et al., 2017) neurotypical children and in samples of autistic children (Lidstone et al., 2014).

We used the original factor analysis subscales (Leekam et al., 2007) which are suitable for young children and include a wider range of items than other published RBQ-2 RSMB and IS subscales. Scores for each 2-factor subscale are averaged across the valid items completed to account for missing data giving potential scores between 1 and 3. Internal reliability was confirmed for this sample: 0.913 for the Total score, 0.859 and 0.882 for the 2-factor item sets respectively as described above.

#### The screen for child anxiety related emotional disorder (SCARED)

The parent version of SCARED is a 41-item questionnaire designed for children aged 7–18 years old (Birmaher et al., 1999), but has also been used for younger children of 4–8 years (Adegboye et al., 2022). Parents select one of three ratings (not true or hardly ever true, somewhat true, or sometimes true, and very true or often true) to describe their child’s anxiety-related behaviors in the last 3 months. The SCARED is a reliable, valid, and sensitive measure of anxiety disorders. Its psychometric properties are well established (Birmaher et al., 1999) and recent research shows measurement invariance, test-retest reliability, and acceptable external validity (Behrens et al., 2019). A score of 0–2 is applied to each item and all item scores totaled to arrive at the Total Score used in the current study (internal consistency 0.940). For missing items, the means were averaged from valid scores. Children with high anxiety scores were identified using the clinical anxiety cut-off score of ≥25.

#### Lucid ability computerized assessment system

Verbal and non-verbal reasoning tasks were selected from the Lucid Ability Computerized Assessment System (Singleton, 2001; GL Assessment, 2014). The Lucid Ability Assessment System has good test retest reliability, internal consistency, and validity. It has been validated against other verbal and non-verbal tests of ability including Weschler Intelligence Scale for Children (WISC-III), British Ability Scales (Second Edition), British Picture Vocabulary Scale (Second Edition), NfER Nelson Verbal and Non-Verbal Reasoning Tests and Matrix Analogies Test. The norms were based on large-scale national standardizations involving over 2,300 children across the age and ability range selected from different parts of the UK, to produce norms representative of the national population across ages. For children aged 4–6 years verbal reasoning is assessed by a picture vocabulary task, and non-verbal ability by a mental rotation task. For older children, aged 7–16 years, verbal ability is assessed *via* a conceptual similarities task, and non-verbal ability through a matrix problem-solving task. See Paine et al. (2021) for a detailed description of selected tasks used in the NDAU protocol. The distribution plots for age are shown in Supplementary Materials (Supplementary Figures 1 and 2). Standardized scores for verbal and non-verbal reasoning were used in analyses.

#### British picture vocabulary scale

The British Picture Vocabulary Scale (Dunn and Dunn, 2009) provided a measure of receptive vocabulary ability. In each trial, children were presented with four pictures. The experimenter said one word aloud, and the child was asked to select the picture that best matched the meaning of the word. Standardized scores were used in the analyses.

#### The strengths and difficulties questionnaire (SDQ)

The SDQ (Goodman, 1997) is a 25-item questionnaire, designed to screen for emotional and behavioral difficulties in children aged 3–16 years. Each item is scored on a three-point scale, where “0” represents “not true,” “1” is “somewhat true,” and “2” is “certainly true.” Its psychometric properties have been extensively tested (Goodman et al., 2000), and population norms are available (Meltzer et al., 2003). Parents were asked to rate their child’s behavior over the last 6 months. In line with scoring guidelines, a total difficulties score was calculated from 20 of the items excluding the prosocial subscale. Four subscales (5 items each) were then analyzed; emotion, conduct, hyperactivity/inattention, and peer relationship. The internal consistency for each scale and total ranged from 0.60 to 0.80, slightly higher than the range found in previous studies (Goodman, 2001; Stone et al., 2015). For missing items, the means were averaged from valid scores.

#### The Welsh index of multiple deprivation (WIMD)

Socio-economic status was assessed using the WIMD which is a measure of deprivation for small areas in Wales from 1 (most deprived) to 1,909 (least deprived). Deprivation indices include income and employment. The range of rank and quintile scores in the sample is shown in Table 1.

### Data analysis plan

Data analyses were carried out using SPSS Version 26 (IBM Corp, 2019). The significance level was defined as *p* < 0.05. Initial data-screening was conducted to assess missing data and the distribution of scores. A missing value analysis using Little’s Missing Completely at Random test was not significant (*p* > 0.05). For RBQ-2, 24 participants (9.2%) were missing one item, and 2 participants (0.8%) were missing two items. For SDQ, 11 participants did not have any data (4.2%), one participant had nine missing items (0.4%), and another participant had 17 missing items (0.4%). For the SCARED, 18 participants did not have any data. Of these four were missing SDQ scores and three were missing a Lucid or SES score. Only the SDQ total was normally distributed; the remaining variables violated assumptions of normality according to Shapiro-Wilk statistic and non-parametric analyses were used to replace parametric analyses as appropriate if results differed.

In the first stage of the analysis, the purpose was to describe the pattern of RRBs in the current sample. Frequency summaries and analyses of mean and medians were conducted for RBQ-2 and for all variables (see Tables s 1–Tables s 4). Population norms and/or data from community samples were also provided for comparison purposes where available (see Tables s 2, 4).

**Table 2:** Frequency of ratings (1 = never or rarely, 2 = mild or occasional, 3 = marked or notable) for each item of the RBQ-2 in the current sample and in a community sample of 6-year-olds (from Uljarević et al., 2017).

Ratings for NDAU sample			Ratings for 77 m-old community sample (Uljarević et al., 2017)		
	1	2	3	1	2	3
1. Arrange toys or other items in rows/patterns?	51.2	39.2	9.6	61.1	38.1	0.8
2. Repetitively fiddle with toys or their items?	33.5	32.3	34.2	67.5	24.6	7.9
3. Spin him/herself around and around?	52.9	28.0	19.1	77.0	19.0	4.0
4. Rock backwards and forwards or side to side,	66.5	17.9	15.6	86.5	11.1	2.4
5. Pace or move around repetitively?	63.0	18.3	18.7	80.2	12.7	7.1
6. Make repetitive hand / finger movements?	62.3	20.8	16.9	84.1	11.1	4.8
7. Have a fascination with specific objects	48.5	28.8	22.7	54.0	34.9	11.1
8. Like to look at objects from particular angles?	66.9	25.7	7.4	72.2	24.6	3.2
9. Have a special interest in smell (objects/people)	67.8	20.2	12.0	80.2	16.7	3.2
10. Have a special interest in the feel of surfaces?	53.3	31.1	15.6	65.1	31.2	3.2
11. Have special objects he/she likes to carry	54.2	23.5	22.3	62.7	22.2	15.1
12. Collects/hoards items of any sort?	53.5	25.6	20.9	46.8	28.6	24.6
14. Get upset about minor changes to objects	54.2	27.3	18.5	77.0	21.4	1.6
15. Insist that daily routines remain the same?	43.8	30.4	25.8	75.4	23.0	1.6
16. Insist on doing things in a certain way	45.8	29.2	25.0	68.2	31.0	0.8
17. Same music, game or video, or book	32.0	41.3	26.6	40.5	53.2	6.3
18. Same clothes or refuse to wear new clothes?	54.1	31.5	14.4	71.4	23.0	5.6
19. Insist on eating the same foods	46.5	26.5	26.5	65.1	25.4	9.5
20. Limited pattern of self-chosen activities	20.8	47.5	31.7	50.1	45.2	4.6

*Note 1: RSMB subscale includes items 1–6 and 8–10. IS subscale includes items 11 and 13–19. Items 7 and 12 are not included in subscales. Total score includes items 1–20 inclusive). For full details of items and their ratings, see Leekam et al. (2007)*.

**Table 3:** Means (SD) and medians (IQR) for total RRB score and RRB subtype scores on RBQ-2 by age (*N* = 260).

RBQ-2 (*N* = 260)			
	Mean (SD)	Median	95% CI for Mean
Total RRB			
4–5 y	1.72 (0.48)	1.65	1.63–1.82
6–8 y	1.67 (0.48)	1.65	1.59–1.74
All	1.69 (0.48)	1.65	1.63–1.75
RSMB			
4–5 y	1.62 (0.53)	1.44	1.52–1.72
6–8 y	1.57 (0.50)	1.44	1.49–1.65
All	1.59 (0.51)	1.44	1.53–1.65
IS			
4–5 y	1.78 (0.62)	1.63	1.66–1.90
6–8 y	1.72 (0.57)	1.63	1.63–1.81
All	1.74 (0.59)	1.63	1.67–1.81

*Note: None of the RBQ-2 variables are normally distributed. RSMB, repetitive sensory motor behavior; IS, insistence on sameness*.

**Table 4:** Correlations between RBQ-2 and the demographic and developmental measures.

	RBQ RSMB	RBQ IS	Total RBQ
RBQ IS	−0.638**	-	
RBQ Total	−0.879**	−0.908**	-
SCARED Total	−0.242**	−0.377**	−0.331**
Sex	−0.185**	−0.168**	−0.210**
Age	−0.109**	−0.113**	−0.131**
WIMD Rank	−0.109**	−0.177**	−0.147**
BPVS Standard	−0.082**	−0.043**	−0.074**
Lucid Verbal Reasoning	−0.020**	−0.079**	−0.043**
Lucid Non-Verbal Reasoning	−0.093**	−0.069**	−0.083**

Note: **p > 0.01. *N* = 260 for correlations with RSMB, IS, Total RBQ, Sex, and Age. *N* = 247 for correlations with WIMD Rank. *N* = 252 for correlations with BPVS. *N* = 256 for correlations with Lucid verbal reasoning. *N* = 251 for correlations with Lucid non-verbal reasoning. RBQ, repetitive behavior questionnaire; RSMB, repetitive sensory motor behaviors; IS, insistence on sameness; SCARED, Screen for Anxiety Related Emotional Disorders; BPVS, British Picture Vocabulary Scale.

In the second stage, associations between RRBs and developmental and demographic variables were examined. Correlational analyses were first used to examine the associations between RRBs (Total and the two RSMB and IS subtypes) and all the variables (age, BPVS, Lucid verbal, Lucid nonverbal, anxiety, sex, SES). Associations between sex and other (continuous) variables were explored by computing a point-biserial correlation (a special case of Pearson’s product moment correlation). Bonferroni correction was applied for multiple comparisons (0.05/9 = 0.005). Regression models were then used to examine the contribution made by the demographic variables (age, sex, SES) and developmental variables (BPVS, Lucid verbal, Lucid non-verbal, anxiety) with each subscale of RRBs (RSMB and IS), to determine the relative influence of these variables. Where predictor variables made a significant contribution to the regression models, follow-up tests were conducted to explore these effects. For the regression models, there was no independence of residuals, as measured by the Durbin-Watson statistic. Homoscedasticity was present, as assessed by visual inspection of a plot of studentized residuals vs. unstandardized predicted values (see Supplementary Figures 3 and 4 of plots of the residuals). There were no studentized deleted residuals greater than ±3 standard deviations, no leverage values greater than 0.2, and values for Cook’s distance above 1.

Finally, correlations were run to explore the association between RRBs and the SDQ subscales (total score, internalizing, externalizing, and four subscales: emotion, peer, conduct, and hyperactivity). With Bonferroni correction applied the *p*-value was 0.05/7 = 0.007.

## Results

Characteristics of the children including all demographic and developmental variables under study; age, sex, SES, verbal, non-verbal ability, and anxiety are shown in Table 1. The majority were 6 years or older (60%) and were male (66.9%). The majority also had verbal and non-verbal ability in the average range (within 1 SD from the mean; BPVS, 68.4%, verbal Lucid, 70.7%, non-verbal Lucid 55.3%) and approximately 70% had anxiety scores in the normative range. The percentage with below-average ability and high anxiety scores is shown in Table 1.

Table 2 shows the distribution of responses for each item of the RBQ-2. Seven of the 20 RBQ-2 items (35%) were endorsed with a rating of 2 or 3 by 50% of the sample. This compared with only 2 (10%) items endorsed by more than 50% of a community sample of 6-year-olds (Uljarević et al., 2017). For every item in the questionnaire, there were more “marked” ratings endorsed by parents for the current sample than for previous community samples (Uljarević et al., 2017). Item scores are summarized as mean and median scores according to RRB subtype and age in Table 3. As shown there, scores for IS were higher than for RSMB (*t*_(259)_ = −5.07, *p* < 0.001), and this difference applied to both older and younger children. The same pattern of findings was found using non-parametric tests (Wilcoxon Signed Ranks test for RSMB and IS differences, Mann-Whitney test for age difference).

**Figure 1:**
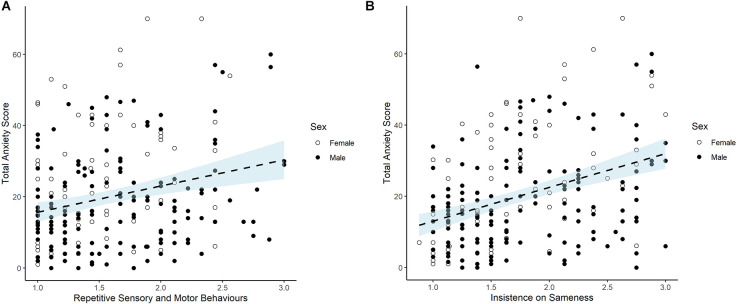
**(A)** RBQ-2 RSMB subscale, Anxiety and Sex. For Scatterplot showing relation between repetitive sensory and motor behaviors score (RSMB) and total anxiety score (measured by SCARED). Note: Each dot represents an individual participant. For RSMB subscale, scores of 1 (never/rarely) to 3 (marked/notable) are averaged across nine items. Note that there is no cut-off score but the 10th percentile and 1 standard deviation from the mean are 2.33 and 2.1 respectively. SCARED scores 0–2 for 41 items produce a Total score of 82. Note that scores of 25 or above represent a clinical anxiety cut off. **(B)** RBQ-2 IS subscale, Anxiety and Sex. Scatterplot showing relation between insistence on sameness score (IS) and total anxiety score (measured by SCARED). Note: Each dot represents an individual participant. For IS subscale, scores of 1 (never/rarely) to 3 (marked/notable) are averaged across eight items. Note that there is no cut-off score but the 10th percentile and 1 standard deviation from the mean are 2.74 and 2.33 respectively. SCARED scores 0–2 for 41 items produce a Total score of 82. Note that scores of 25 or above represent a clinical anxiety cut off.

Correlations (Table 4) were run to explore associations between RRBs and developmental and demographic factors reported in the literature as associated with RRBs. Spearman’s correlations showed the same pattern of findings as shown for Pearson’s. Results showed that children with higher RRB scores had significantly higher levels of anxiety and males had higher RSMB scores than females. A trend towards significance was found for the IS subtype and sex (*r* = −0.168, *p* = 0.007). The RRB mean score for males was 1.65 (0.53) compared with 1.46 (0.46), for females, and the mean IS score for males was 1.81 (0.59) compared with 1.61 (0.57) for females. Mann Whitney tests found significant sex differences at *p* = 0.003 for RSMB and *p* = 0.007 for IS. As females (*M* = 22.68, SD = 16.71) had higher anxiety scores than males (*M* = 18.44, SD = 13.57; *t*_(240)_ = −2.131, *p* = 0.034) while males had higher RRB scores than females (see Figure 1), partial correlations were conducted to test for the association between anxiety and RRB while controlling for sex. The partial correlation coefficients were significant (RSMB-subscale, *r* = 0.284, *p* < 0.001; IS-subscale, *r* = 0.399, *p* < 0.001). In addition to the correlations above, Table 4 shows that the IS subscale alone was significantly associated with SES (higher IS scores associated with greater ranked deprivation).

A hierarchical linear regression revealed that only sex and anxiety significantly contributed to the RSMB subscale, *F*_(7, 210)_ = 4.711, *p* < 0.001, *R*^2^ = 0.136, adjusted *R*^2^ = 0.107. Table 5 shows that anxiety made the largest contribution (*B* = 0.282) whereby higher anxiety scores predicted higher incidences of RSMB. Males (*B* = 0.191) had higher incidences of RSMB. The hierarchical regression for the IS subscale was also significant, *F*_(7, 210)_ = 0.9067, *p* < 0.001, *R*^2^= 0.232, adjusted *R*^2^ = 0.206. Sex (*B* = −0.184), SES (*B* = −0.167), verbal reasoning (*B* = −0.145), and anxiety (*B* = 0.394) significantly contributed to the final model. With respect to the IS subtype, like the pattern observed for RSMB, males and higher anxiety scores were associated with higher incidences of IS. Lower SES, as measured by WIMD rank, and poorer scores on the Lucid verbal reasoning task were associated with higher reported IS behaviors. However, follow-up partial correlations showed that while the IS-anxiety relation remained significantly high when both SES was controlled (*r* = −0.392, *p* = 0.000) or when Lucid verbal was controlled (*r* = −0.375, *p* = 0.000), partial correlations were not significant for either the IS-SES relation or for the IS-Lucid verbal relation when anxiety was controlled (Bonferroni correction of 0.005, applied).

**Table 5:** Regression analysis of RRBs with demographic and developmental variables.

RSMB				
Step 1	B	SE	*B*	*p*
Age	−0.001	0.003	−0.031	0.638
Sex	−0.201	0.070	−0.191	0.005**
SES	−0.000	0.000	−0.127	0.058
*R* ^2^	0.058			
Step 2				
Age	−0.002	0.003	−0.042	0.589
Sex	−0.243	0.070	−0.231	0.001**
SES	−0.000	0.000	−0.108	0.100
BPVS	−0.001	0.003	−0.020	0.779
Lucid Verbal	−0.000	0.003	−0.010	0.889
Lucid Non-Verbal	−0.001	0.002	−0.021	0.790
SCARED	−0.010	0.002	−0.282	<0.001**
*R* ^2^	0.136			
IS				
Step 1	B	SE	*B*	*p*
Age	−0.003	0.003	−0.058	0.386
Sex	−0.175	0.079	−0.147	0.028*
SES	−0.000	0.000	−0.182	0.007**
*R* ^2^	0.064			
Step 2				
Age	−0.003	0.003	−0.061	0.412
Sex	−0.219	0.075	−0.184	0.004**
SES	−0.000	0.000	−0.167	0.007**
BPVS	−0.003	0.003	−0.073	0.285
Lucid Verbal	−0.006	0.003	−0.145	0.034*
Lucid Non-Verbal	−0.001	0.002	−0.034	0.652
SCARED	−0.015	0.002	−0.394	<0.001**
*R* ^2^	−0.232			

Note: *p < 0.05, **p < 0.01. RSMB, repetitive sensory motor behaviors; IS, insistence on sameness; BPVS, British Picture Vocabulary Scale; SCARED, Screen for Anxiety Related Emotional Disorders.

**Figure 2:**
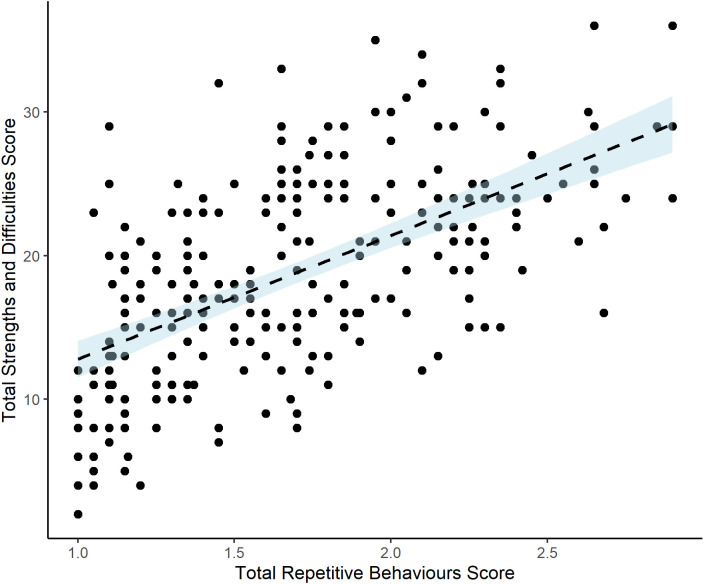
Repetitive behaviors and emotional and behavioral difficulties. Scatterplot showing relation between total repetitive behavior score and total strengths and difficulties score. Note: Each dot represents an individual participant. For RBQ-2 Total Score, scores of 1 (never/rarely) to 3 (marked/notable) are averaged across 20 items. Note that there is no cut-off score but the 10th percentile and 1 standard deviation from the mean are 2.35 and 2.17 respectively.

Finally, further analyses explored the relation between RRBs and the emotional and the behavioral profile of this sample using the parent SDQ. Table 6 shows the mean SDQ scores for the NDAU sample. These were twice as high as the population means. No sex differences emerged for the SDQ. Table 7, Figure 2, and Supplementary Figures 5 and 6 show the correlations between RRB and SDQ total score and each of the subscales. For the SDQ Emotion subscale, which was expected to be aligned with anxiety, significant coefficients of 0.272 and 0.341 were found. Highly significant correlations were also found between RRB and all other SDQ subscales.

**Table 6:** Means (SD) of SDQ scores and percentage of sample with highly elevated scores (*N* = 260).

	Mean (SD)	Percent with high/very high SDQ scores	Population sample for SDQ (*n* = 5855) Mean (SD)
SDQ Total	18.74 (7.07)0	59.2	8.6 (5.7)
Emotion	3.60 (2.61)	32.4	1.9 (2.0)
Conduct	4.27 (2.73)	56.7	1.6 (1.7)
Hyperactivity	7.69 (2.54)	63.6	3.6 (2.7)
Peer	3.19 (2.33)	42.1	1.4 (1.7)
Internalizing	6.78 (4.01)	-	-
Externalizing	11.96 (4.45)	-	-

*Note: Population data is based on a large representative sample of British children between the ages of 5 and 10 (see Meltzer et al., 2003). No norms were calculated for internalizing and externalizing scores*.

**Table 7:** Correlations between RBQ-2 and SDQ measures.

	RBQ RSMB	RBQ IS	Total RBQ
SDQ Emotion	0.288**	0.379**	0.360**
SDQ Conduct	0.314**	0.334**	0.361**
SDQ Hyperactivity	0.479**	0.320**	0.414**
SDQ Peer	0.480**	0.482**	0.519**
SDQ Total	0.555**	0.541**	0.592**
Internalizing	0.474**	0.530**	0.543**
Externalizing	0.466**	0.382**	0.457**

*Note: *N* = 260 for correlations with RSMB, IS, and Total RBQ. *N* = 247 for correlations with all SDQ scales. SDQ, Strengths and Difficulties Questionnaire; RBQ, Repetitive Behaviors Questionnaire; RSMB, repetitive sensory motor behaviors; IS, insistence of sameness*.

## Discussion

Using a transdiagnostic approach, the current study examined RRBs in a diverse sample of children in relation to a range of developmental and demographic correlates. Traditionally RRBs have been researched through a lens focused on specific diagnosed groups (e.g., ASD) or else on matched neurotypical control groups or on community samples. Although children in the current sample were recruited for the purpose of assessing and supporting their difficulties at school, unlike recruitment for most studies, they were not selected according to membership of a clinical category. Setting aside diagnostic categories gave us the opportunity to produce evidence documenting the range of variation of RRB in a sample of this kind for the first time.

First, we found that parents of children in this sample frequently endorsed extreme scores for their children. It can be seen from Table 2 that children from the current sample showed more “marked” or “notable” scores than seen in a community sample of 6-year-old children (Uljarević et al., 2017). In terms of mean scores (Table 3), the current sample had a total mean RRB score of 1.69 (SD 0.48) which is higher than in the Uljarević study (mean 1.36 (SD 0.39) but lower than scores reported for autistic children [e.g., Lidstone et al., 2014, mean 1.96 (SD 0.41)]. Thus, children in the current sample who are likely to be at risk for a range of psychological conditions fall along a continuum between the community sample and autistic individuals in terms of their levels of repetitive behaviors.

Second, this research contributed new findings relevant to the developmental theory of RRB (Evans et al., 1997, 1999; Leekam et al., 2011). The developmental theory proposes that elevated levels of RRBs are initially adaptive for neural and motor development and reduce in favor of self-regulated actions as children age. Excessive RRBs maintained at older ages may therefore signal delay or difficulty with regulatory functions. In line with this proposal, with respect to cognitive and language domains, past evidence shows high levels of RRBs associate with lower developmental language and cognitive level (Harrop et al., 2014; Larkin et al., 2017) and in the emotion domain, high levels of RRBs associate with high levels of fears and anxiety (Rodgers et al., 2012b; Lidstone et al., 2014). The correlational and regression results in this study partially supported these earlier findings (see Tables s 1, 5). In terms of language and cognition, RRB did not significantly associate with non-verbal ability, or with language except for a small effect of one language measure for the IS subtype only. In contrast, anxiety was correlated with both subtypes of RRB and made the largest contribution to RRB of any variable in the regression model.

The research also contributed to recent debates about the subtype-specificity of RRB particularly the specificity of the IS subtype in relation to anxiety (Sellick et al., 2021). The current study did not find evidence for this. Instead, higher anxiety scores were associated with higher scores on both IS and RSMB subtypes. Similarly, we did not find a selective association between RSMB and language and cognitive variables as previously found by Larkin et al. (2017). Of the participant characteristics that might explain these current findings, the most relevant might be the age range or developmental level of the sample. Selective associations between RSMBs and language and cognitive ability may have been found previously as children, at age 2–3 years were at a developmental level at which RSMB were high but IS behaviors had not yet fully emerged. By the beginning of the school years from age 4 to 5 years, language and cognitive skills have stabilized for most children, while RSMBs have decreased and are potentially less relevant to higher-level cognition and language. In contrast, as shown by our results of higher IS than RSMB scores and negative correlation between IS and age (Tables s 3, 4), IS behaviors peak at around 4 years and tend to reduce to some extent after that, coinciding with the age when common childhood fears and anxiety are also increasing (Evans et al., 1999), and so the association between RRB and anxiety may be particularly high at this age. Further research involving different samples will be needed to further investigate this proposal.

Analysis of the demographic variables also helped to address debates in the literature about the role of sex in ASD given previous mixed findings. The results of the current study showed that males had higher RRB scores than females for both RRB subtypes and that sex made a significant contribution to the regression model. These results add to the limited body of evidence showing higher RRB scores in males against a background of studies that have mostly not reported a sex difference (see Uljarević et al., in press for review). Follow-up analyses showed that although males had higher RRB scores than females, females had higher anxiety scores than males and that when sex was controlled for in a partial correlation analysis, the relation between RRB (both subtypes) and anxiety was still strong. This suggests that the association between RRB and anxiety is not primarily driven by the higher RRB scores of males. However, further research on RRB and sex differences is needed given the unequal sample size in the current study.

The relation between higher IS scores and greater ranked socio-economic deprivation also needs further interpretation. To our knowledge, a similar finding has been reported in only one study. That study, with younger children, also sampled participants with low SES levels (Leekam et al., 2007; Larkin et al., 2017) and associations were found with RSMB instead of IS. However, with older children, an environment associated with financial and social deprivation might foster higher levels of insistence on sameness as a way a child can attempt to structure uncertain experiences in their home environment. The contribution of SES to the anxiety-IS relation might be complex and include other variables beyond those included in the current study. For example, Larkin et al.’s (2019) longitudinal study has shown the importance of maternal depressive symptoms in relation to RRB outcomes in younger children. Hence we might conjecture that heightened IS and heightened anxiety in children in this sample may be related to SES *via* the familial and social environment. Further analysis is beyond the scope of the current study but deserves future research attention.

The findings also reveal previously unacknowledged correlates of RRB. This sample was recruited to assess and give support to behavioral, emotional, and cognitive difficulties and it offered the opportunity to explore whether RRBs may be associated with other factors beyond those previously studied. To do this we looked at parent ratings on the SDQ. In the current study, moderate to high correlations were found between each RRB subtype and SDQ scores, including those for internalizing scale (items from the emotion and peer relations subscales), externalizing scale (items from the conduct and hyperactivity subscales), and each of individual subscales (Table 7 and Figure 2). The results closely replicate the findings of Ghanizadeh and Moeini (2011), conducted in a different country, with a different type of sample (preschool children recruited from a community sample), and assessed with a different RRB measure. These converging findings strongly support the view that children’s RRBs across different populations are correlated with broader indices of mental health and well-being than have previously been considered. The similar correlation coefficients for the SCARED, a specific anxiety measure, and for the more general SDQ emotion subscale also support results by Bryant et al. (2020), of convergence between results on the SDQ emotion sub-scale and another specific anxiety scale in a separate transdiagnostic sample.

This research has important limitations regarding its design and methodology. It includes only a single measure of RRB and a single parent informant. In addition, it includes a single and distinctive sample referred for a particular purpose and a sample with a strong sex imbalance, an age range limited to the early school years, and a low SES. All these factors are dangers to the generalizability of the study. However, many previous studies of small samples also lack generalizability in comparison to the current sample, because participants are seldom recruited from lower SES environments. A further consideration is whether the study should have included an assessment of ASD symptoms to clarify the interpretation of the results. If high numbers of children in this sample had elevated autism traits, indicating a likelihood for a future diagnosis of ASD, this could be seen to undermine the distinctiveness of a proposed transdiagnostic approach. On the other hand, the purpose of the study was to focus exclusively on describing only one ASD-related domain, RRBs in children that had not been selected with high levels of RRBs as part of their diagnosis and this purpose was met. Nevertheless, further research is clearly needed to identify the role of the other ASD domain, social communication, in the RRB-anxiety relation. Meanwhile, the results to date serve best as pointers towards new directions for the future of RRB research and raise several questions for future attention. For example, new research studies will help us understand more about anxiety and mental health in relation to RRBs and whether social communication difficulties play a role in this relation, irrespective of ASD diagnosis.

The strong association between RRBs and the measures of mental health raises the question of whether RRBs might be a proxy for a broader construct of psychopathology instead of being a distinct and specific construct. This would be consistent with (Lahey et al., 2017a) hypothesis of a general factor of psychopathology on which lower-order factors load. A bifactor model would reflect both a general factor and more specific subfactors. Several studies of psychiatric disorders in adolescents investigating the bifactor model have shown evidence for a general psychopathology factor (Caspi et al., 2014; Patalay et al., 2015). Likewise, research using the SDQ subscales also found the best fitting model was a bi-factor model with externalizing and internalizing as two global factors (Caci et al., 2015). Although at this stage, the purpose of the research is to clarify the contribution of developmental and demographic correlates of RRB in a diverse sample, further research focusing exclusively on the SDQ and RRB measures is needed to examine this proposal more directly. One consideration is that the classic definition of psychopathology may not easily apply to RRBs given that some RRBs may be adaptive, contribute to the development, or help enhance life functions for the individual. However, future research with representative samples will be particularly important (Lahey et al., 2017b) to explore this. To date, one published study has used a representative sample but did not show strong measurement overlap between RBQ-2 and SDQ, among a special needs “at risk” group (Wigham et al., 2012) and inspection of the scatterplots (Figures 1, 2) in the current study at the higher scoring end, supports that result. However more empirical work is needed, especially research focusing on RRB and the externalizing and internalizing factors as identified by Caci et al. (2015).

Another question for future research relates to developmental explanations of RRB and the role played by language and cognition. While our results showed that language and cognitive skills are not strongly associated with RRB, only structural aspects of language were analyzed rather than communicative aspects of language more generally. Given that the interdependence of social communication and RRB is a necessary condition for a diagnosis of ASD, future research needs to focus on the social pragmatics of language separately from structural language. The strong correlation we found between RRBs and the SDQ peer relations scale gives further support for a relation with social aspects of language. Further examination of the relation between RRBs and language/cognition is also needed for neurodevelopmental populations (e.g., genetic conditions, ASD) that are characterized by developmental delay as part of their diagnostic criteria, compared with those without early language or cognitive delay.

Finally, the results open the potential to rethink previous concepts and assumptions regarding RRBs. One question is whether the conceptualization and measurement of RRB which is drawn from developmental psychology theory but most heavily influenced by research on ASD, best represents children’s development more broadly regardless of children’s clinical label or category. As Burack et al. (2021) point out, “when viewed in the context of disorders such as ASD, repetitive behaviors are too often seen as mere symptoms, rather than as tools for adaptation.” A developmental perspective of RRBs in terms of adaptation (Evans and Gray, 2000; Evans et al., 2014) views RRBs as serving to regulate emotion, sensation, and/or information processing even if the child is not using the most developmentally appropriate or optimal self-regulatory strategies. Therefore, RRBs may have an adaptive purpose even when self-regulatory strategies are developmentally delayed, impaired, or overwhelmed for any reason. Future conceptual and empirical work should clarify the adaptive nature of RRBs in terms of how they serve particular functions for an individual child while at the same time impacting the child’s development progress in different ways. For example, regardless of age and developmental level repetitive motor stereotypies may serve an important function of regulating sensory stimulation. At the beginning of life, these behaviors are also strongly developmentally adaptive in serving neural and motor development, but at later ages, this developmental function subsides. Similarly, regardless of age and developmental level, insistence on sameness in routines and environment functions to regulate emotion but developmental changes enable alternative flexible thinking and behavior which serve the complementary function to regulate emotion. Because behavior patterns themselves contribute to the experience and developmental change, it may be helpful for the clinical and educational practitioners to keep in mind these different interpretations of adaptation while aiming to respect the benefits of RRBs while also supporting the benefits of enhancing behavioral flexibility and variety, depending on the individual’s developmental potential. Support at an individual level could also take account of sex-specific adaptive strategies in males compared with females and the effect of the impact of RRBs on family life, rather than on simply the presence of behaviors in the individual.

To conclude, despite its limitations, this study provides the first evidence of RRBs in a diverse sample of this kind. The results support evidence that irrespective of the diagnostic status and nature of the specific population (clinical vs. non-clinical), anxiety serves as a crucial correlate, and potential mechanism, behind diverse RRB expressions. Importantly, the study contributes new evidence about other lesser-known correlates of RRB. The clear associations between RRBs and the SDQ subscales including conduct, hyperactivity, and peer relations as well as emotion, indicate that it is time to move on from traditional approaches to RRBs. While our results confirm the significance of emotional difficulties for RRB in the early childhood years, a broader interpretation of RRBs is needed beyond existing clinical and developmental explanations. We conclude that children’s RRBs are best represented on a continuum of severity incorporating all populations and that repetitive behaviors in these populations in the early school years are associated not only with anxiety but with broad indices of mental health and well-being.

## Data availability statement

The original contributions presented in the study are included in the article/Supplementary-material, further inquiries can be directed to the corresponding author.

## Ethics statement

The studies involving human participants were reviewed and approved by EC.16.10.11.4592GR. Written informed consent to participate in this study was provided by the participants’ legal guardian/next of kin.

## Author contributions

SL and JK designed the study. SL led the article preparation. JK curated the data and carried out the statistical analysis. MU and DH contributed to the analysis plan and interpretation. SG led all aspects of the sample recruitment and data collection. JK, SL, MU, DH, and SG contributed to the manuscript drafts. SL, SG, and DH contributed to funding acquisition. All authors contributed to the article and approved the submitted version.

## Footnotes

^1^ https://www.cardiff.ac.uk/neurodevelopment-assessment-unit
