# Supplementary material for: Restricted and repetitive behaviors and their developmental and demographic correlates in 4–8-year-old children: A transdiagnostic approach
Source: Front Behav Neurosci. 2023 Mar 1;17:1085404. doi: 10.3389/fnbeh.2023.1085404 (PMC10014551; doi:10.3389/fnbeh.2023.1085404)
Supplement: Supplementary file 1 [file Table_1.DOCX]

Supplementary Material

Restricted and repetitive behaviors and their developmental and demographic correlates in 4-8-year-old children: a transdiagnostic approach

Jennifer Keating, Stephanie Van Goozen, Mirko Uljarevic, Dale Hay, Susan R Leekam*

*** Correspondence:** Susan R Leekam. E-mail address: LeekamSR@cardiff.ac.uk

# Supplementary Figures and Tables

## Supplementary Figures

**
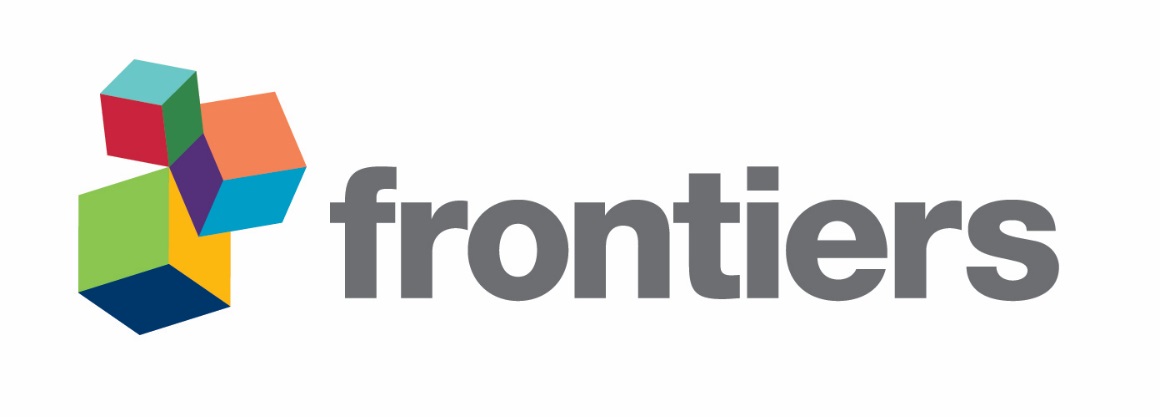
**


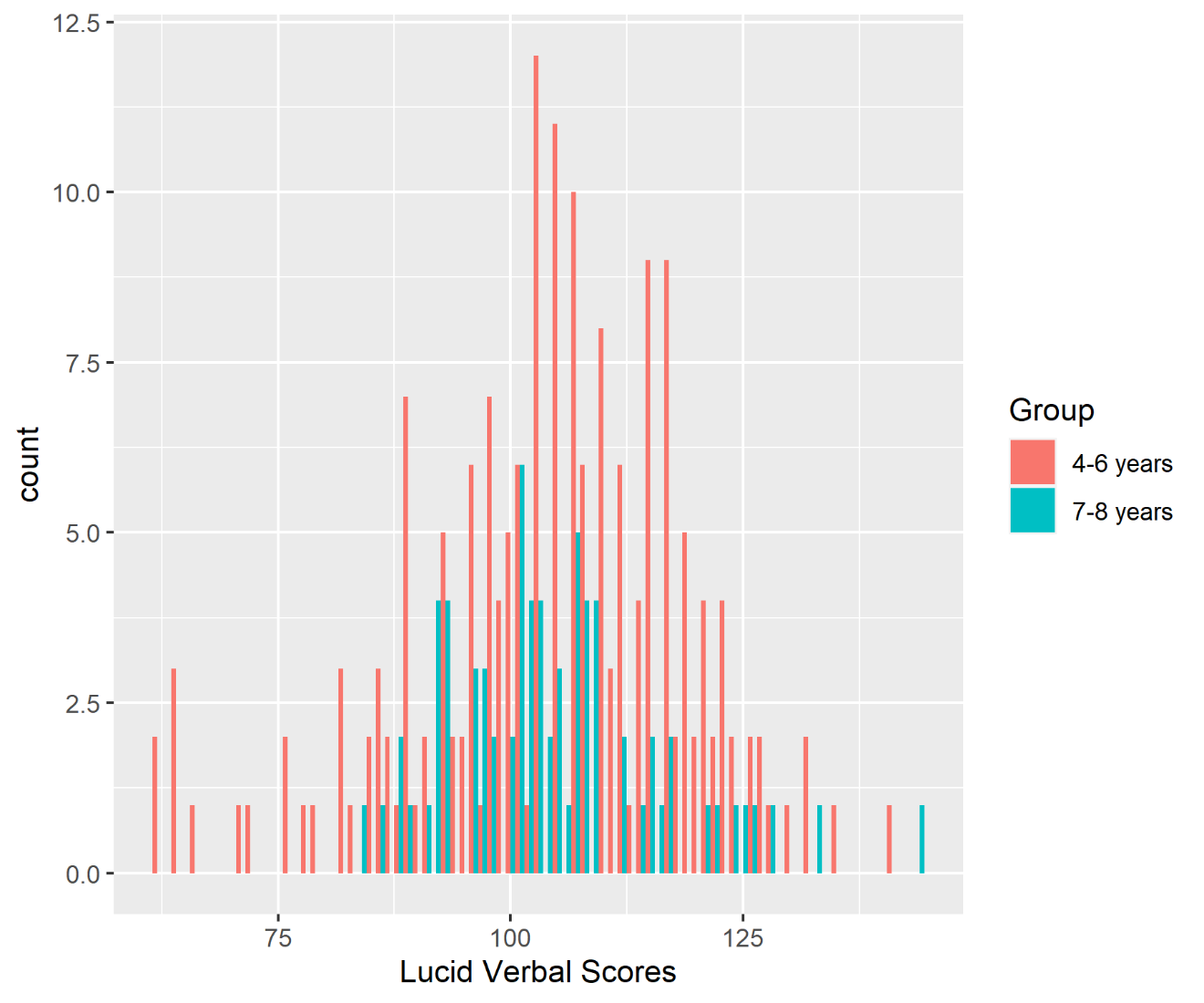


**Supplementary Figure 1.** Distribution of Lucid verbal scores across both age groups. Note that the age groups for Lucid refer to the two different tasks completed (one for 4-6 year olds, another for 7-12 year olds). These do not correspond to the age groups in Table 1.


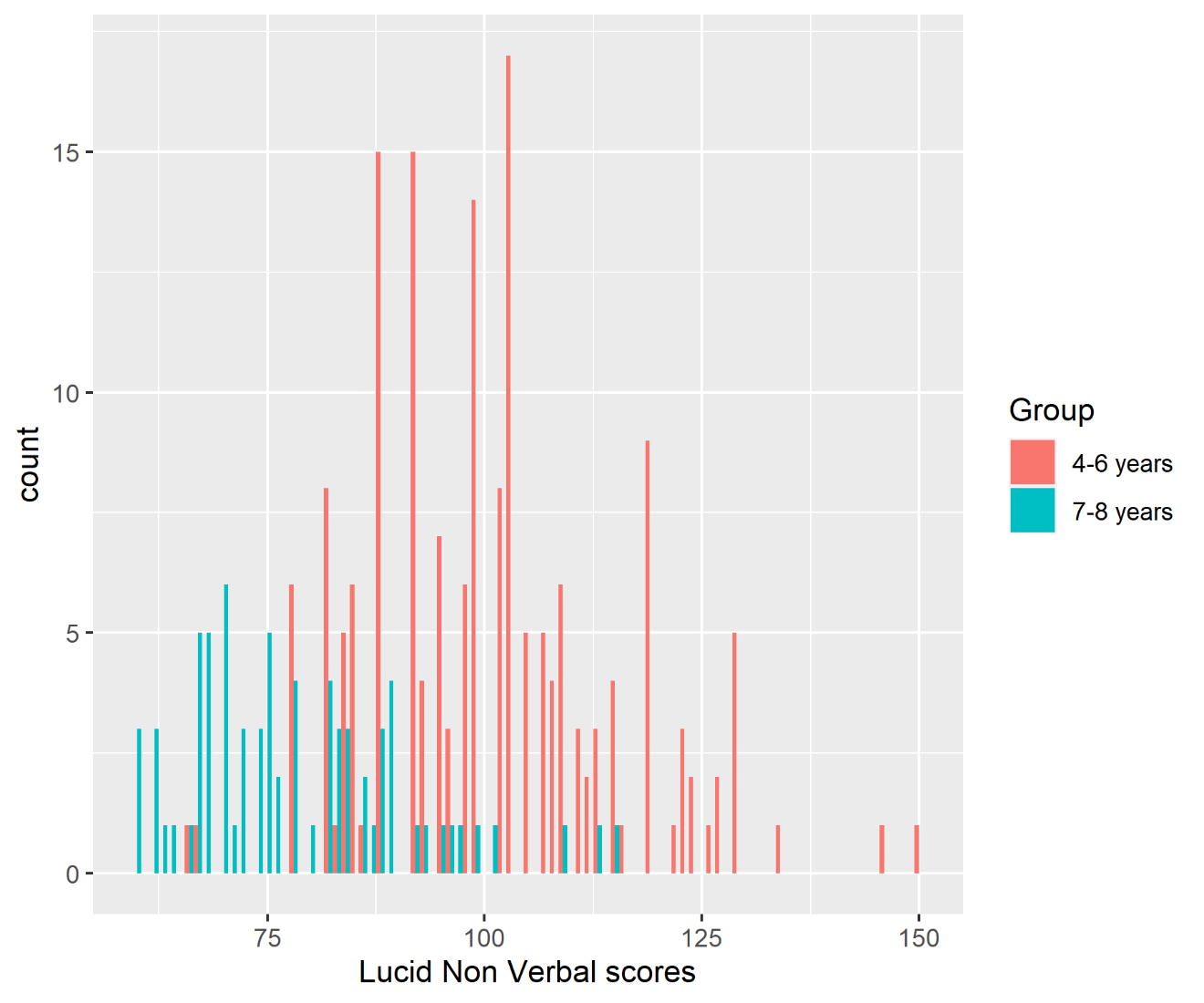


**Supplementary Figure 2:** Distribution of Lucid Non verbal scores across both age groups. Note that the age groups for Lucid refer to the two different tasks completed (one for 4-6 year olds, another for 7-12 year olds). These do not correspond to the age groups in Table 1.


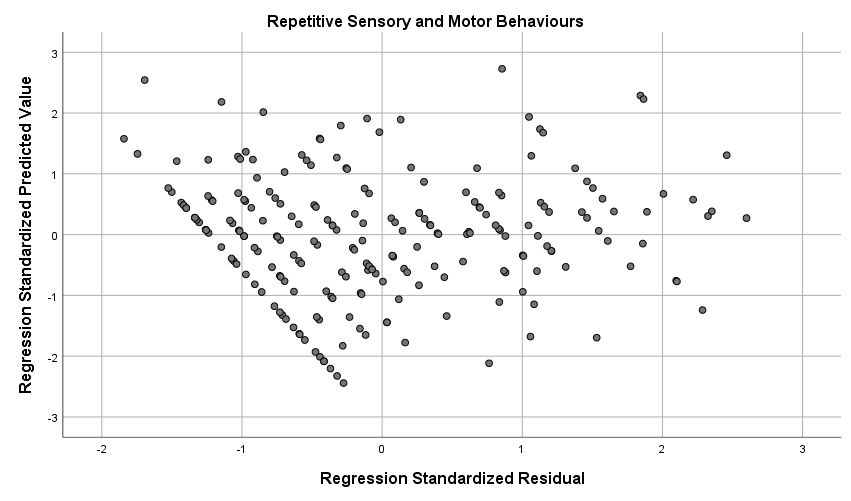


**Supplementary Figure 3:** Plot of studentized residuals versus unstandardized predicted values for regression with repetitive sensory and motor behaviours as dependent variable


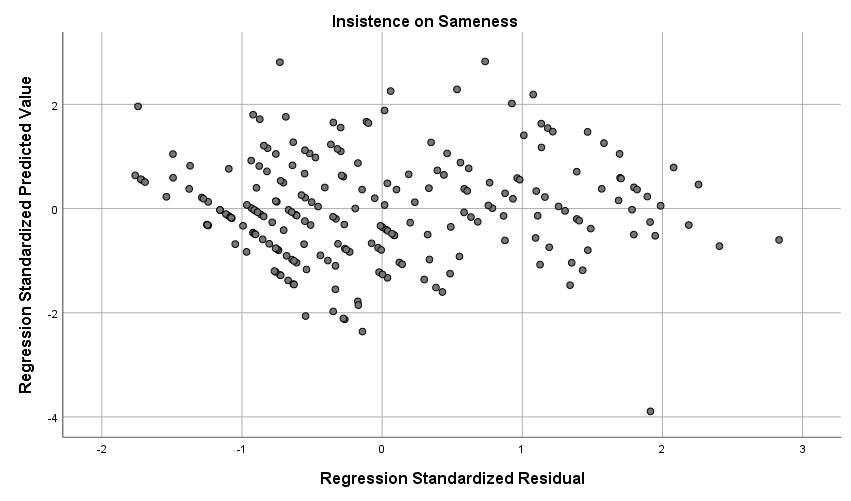


**Supplementary Figure 4:** Plot of studentized residuals versus unstandardized predicted values for regression with insistence on sameness as dependent variable


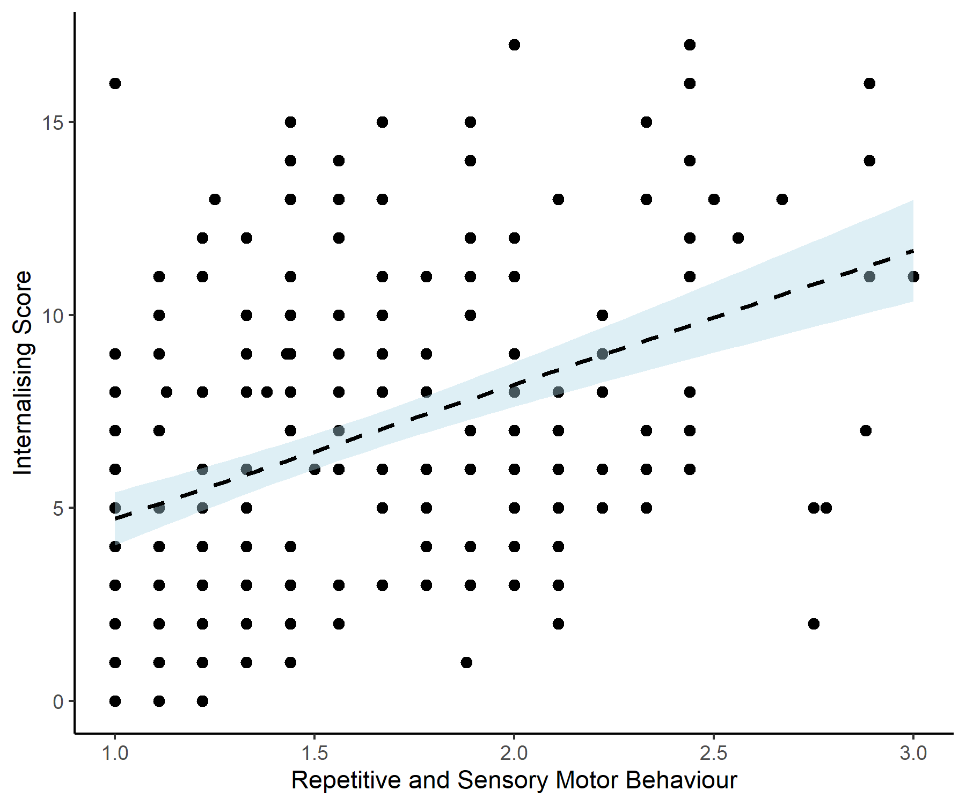


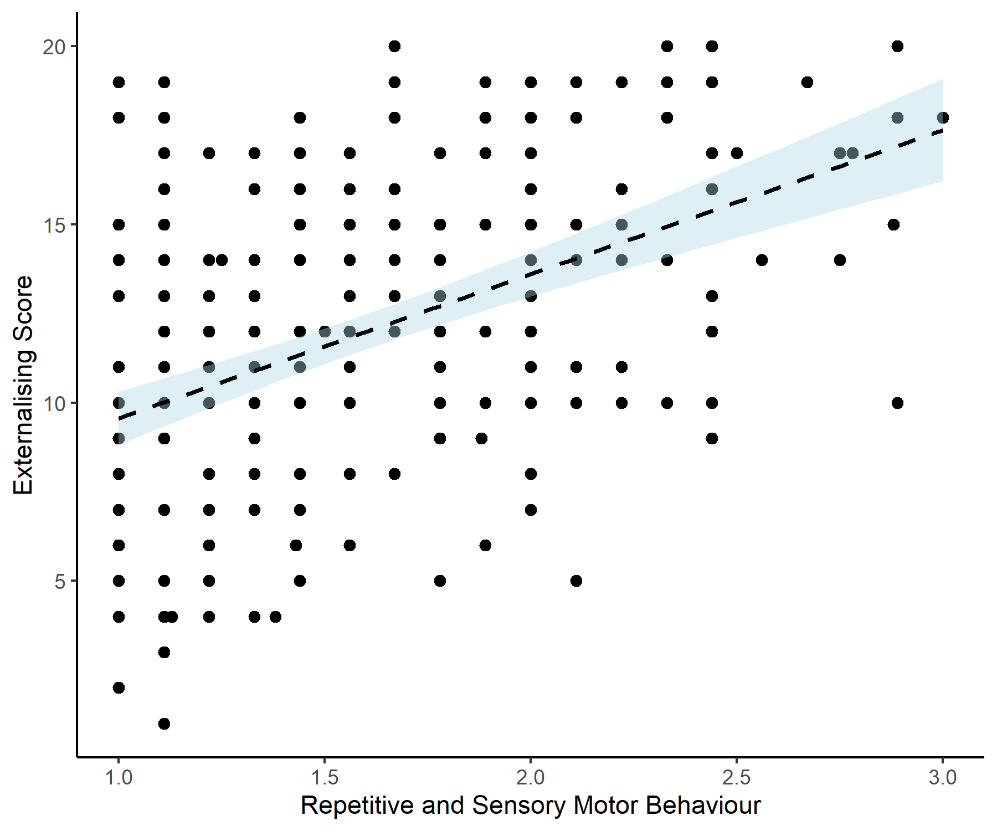


**Supplementary Figure 5:** Scatterplot showing relation between repetitive sensory and motor behaviour score and internalising and externalising scores on the Strengths and Difficulties Questionnaire.
Note: Each dot represents an individual participant.


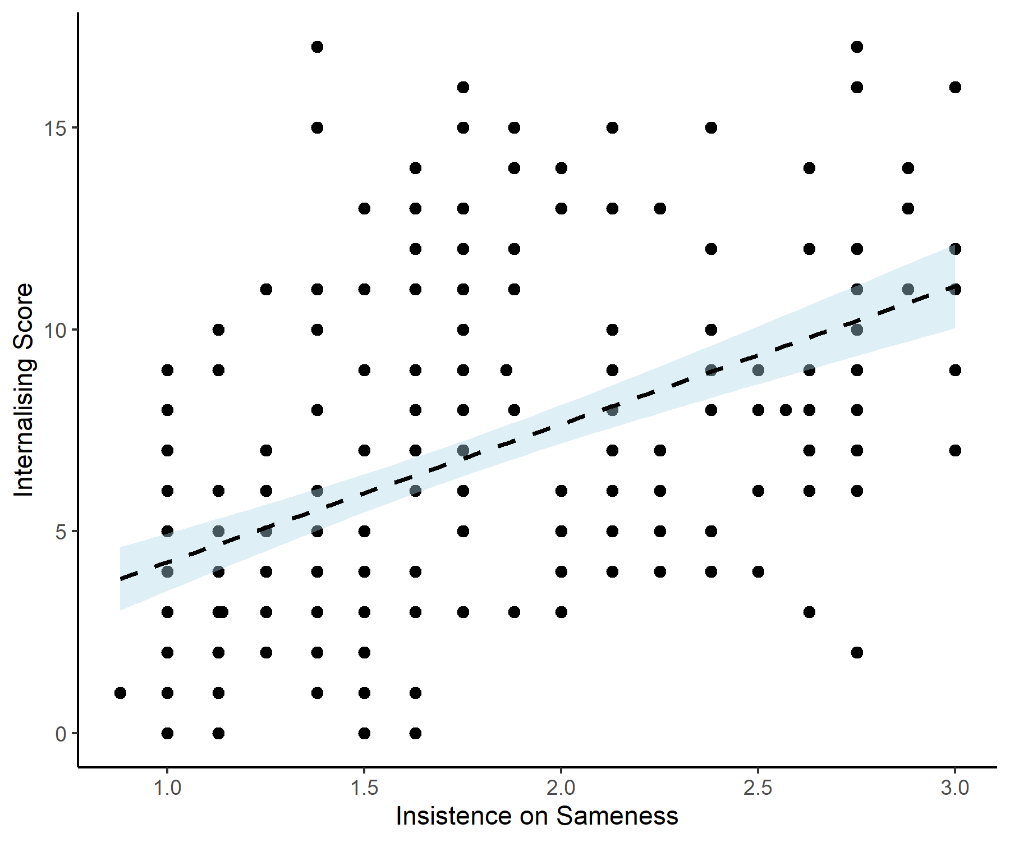


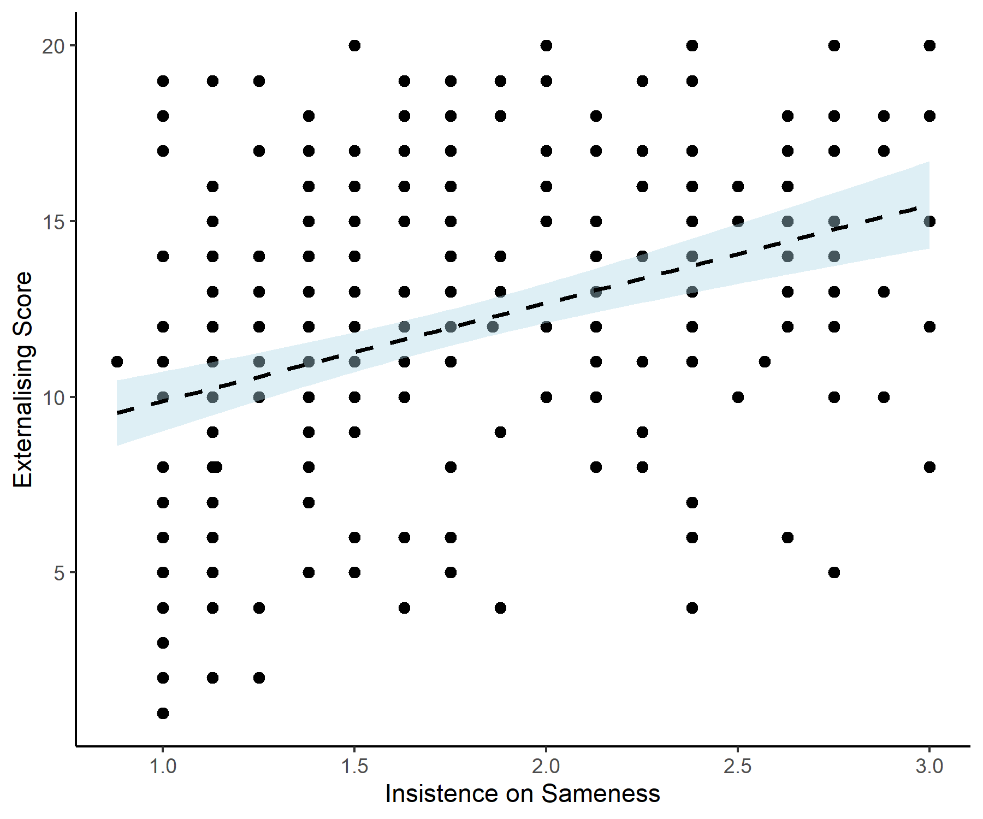


**Supplementary Figure 6:** Scatterplot showing relation between insistence on sameness score and internalising and externalising scores on the Strengths and Difficulties Questionnaire.
Note: Each dot represents an individual participant.
